# Synthesis of Imidazole-Based Molecules under Ultrasonic Irradiation Approaches

**DOI:** 10.3390/molecules28124845

**Published:** 2023-06-19

**Authors:** Xian-Long Yu, Yu-Han Fan, Xu-Nan Zheng, Jing-Fei Gao, Li-Geng Zhuang, Yang-Ling Yu, Jing-Hui Xi, Da-Wei Zhang

**Affiliations:** 1 College of Chemistry, Jilin University, Changchun 130012, China; yuxl8220@mails.jlu.edu.cn; 2 College of Plant Science, Jilin University, Changchun 130062, Chinahxi1965@jlu.edu.cn (J.-H.X.); 3 School of Pharmaceutical Sciences, Jilin University, Changchun 130021, China; fanyh2820@mails.jlu.edu.cn

**Keywords:** imidazole, sonochemistry, synthesis, green chemistry

## Abstract

Imidazole-based compounds are a series of heterocyclic compounds that exhibit a wide range of biological and pharmaceutical activities. However, those extant syntheses using conventional protocols can be time-costly, require harsh conditions, and result in low yields. As a novel and green technique, sonochemistry has emerged as a promising method for organic synthesis with several advantages over conventional methods, including enhancing reaction rates, improving yields, and reducing the use of hazardous solvents. Contemporarily, a growing body of ultrasound-assisted reactions have been applied in the preparation of imidazole derivatives, which demonstrated greater benefits and provided a new strategy. Herein, we introduce the brief history of sonochemistry and focus on the discussion of the multifarious approaches for the synthesis of imidazole-based compounds under ultrasonic irradiation and its advantages in comparison with conventional protocols, including typical name-reactions and various sorts of catalysts in those reactions.

## 1. Introduction

Imidazole is an aromatic nitrogen heterocycle, classified as a diazole, which is the constituent of several natural products such as histamine, nucleic acids, some alkaloids, etc. The specific framework and environmentally exciting rich electrons in the aromatic cycle make imidazole-based compounds exhibit many unique chemical and physical properties. The preceding literature has proven that imidazole derivatives showcase a massive utility in biochemistry, coordination chemistry, material science, industrial applications, and pharmaceutical chemistry [1,2,3,4,5,6]. Particularly, myriad imidazole compounds demonstrate vast biological and pharmacological activities including antibacterial, anticancer, antifungal, and analgesic, indicating imidazole derivatives to be promising small molecular drugs or lead compounds (Table 1). 

**Scheme 1:**
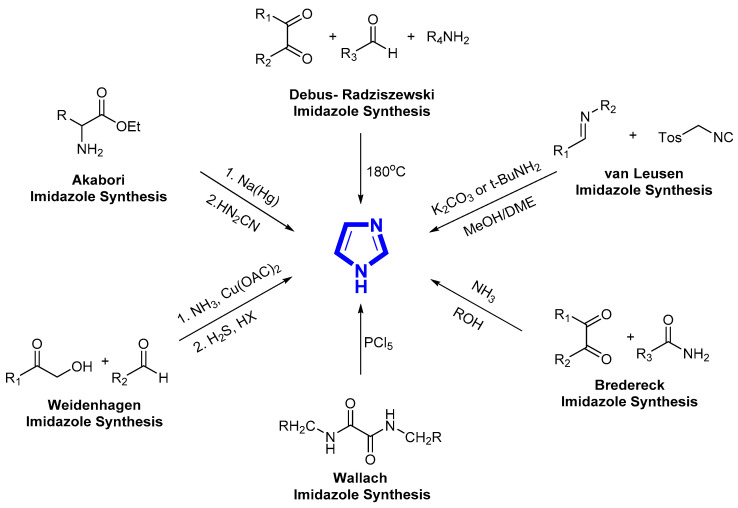
Typical synthetic methods for imidazole and its derivatives.

The history of imidazole synthesis can be traced back to the 1850s [17]. In the past 165 years, numerous reactions and methods for the formation of imidazole scaffolds have been documented, including several well-known methods such as the Debus–Radziszewski reaction [17,18], the Phillips–Ladenburg reaction [19,20,21,22,23], the Wallach reaction [24,25], the Weidenhagen reaction [26], the Bredereck reaction [27], the Akabori reaction [28], and the van Leusen reaction [29,30] (Scheme 1). However, many conventional synthesis methods still have a few drawbacks, which are typically accompanied by harsh conditions, protracted reaction periods, and unsatisfactory yields.

Gargantuan contributions to imidazole derivatives’ synthesis were made by former trailblazers, leading those precursory strategies showing beneficial applications in pharmacy, organic chemistry, and material chemistry as well. Those classic trails, however, are faced with an increasing challenge since their drawbacks, for instance, pollution and low yield, need to be ameliorated in certain ways in order to keep pace with green chemistry ideology as well as cost reduction. 

Consequently, a slew of emerging techniques, such as ultrasound, microwave, and radiant, have burgeoned on account of researchers painstakingly working hard to tackle the shortcomings mentioned. Amongst them, the utilization of sonochemistry has been receiving more attention from researchers as several predicaments are overcome by highly frequent mechanical waves. In other words, the simplification of tedious procedures, the trim of irrelevant reactions, and milder requisites for activating molecules are achieved by harnessing ultrasound as assistance. Eminent advantages of sonochemistry over traditional ways have successively stirred organic, pharmaceutical, and materials chemists’ emotions to delve into its prospective application in synthetic approaches as well as making it a trend.

The ultrasonic irradiation reaction mixture produces a large number of cavitation bubbles, which grow rapidly and then collapse violently, forming microjets and producing fine emulsions between the reactants. Afterward, the collapse of the cavitation bubbles also increases the local temperature inside the reaction mixture and eventually causes the reaction mixture to pass through the activation energy barrier. 

As a consequence of the principle that ultrasound is produced by a process converting mechanical or electrical energy into sound energy, the transferring device is obviously of vital importance [31]. This is the so-called ultrasonic transducer, and there are mainly three types of these: the liquid-driven type, the magnetostrictive type, and the piezoelectric type. The piezoelectric type, constructed of piezoelectric ceramic, is most prevalently utilized in the laboratory [32]. Furthermore, ultrasonic cleaning baths, ultrasonic horns, and probe systems are the most common devices for ultrasonic sources. Hitherto, the ultrasonic cleaning bath is the most widely spread and economical source of ultrasonic irradiation for chemical laboratories. In addition, the ultrasonic probe makes acoustic energy transfer directly into the system without the loss of energy. 

The development of sonochemistry is not a walk in the park but the effort of several generations of researchers. The key point was first reported by Sir Galton, a British physiologist, who invented a silent whistle that is able to emit high-frequency waves, widely named ultrasound. It is virtually certain that the discovery of piezoelectricity by physicists Jacques Curie and Pierre Curie leads to the attainment of generating ultrasound in a water medium [33,34]. Albeit ultrasound was initially engaged in a sonar system for detecting objects, the cavitation phenomenon was observed by Sir Thornycoft and Barnably when noticing the erosion of their submarine propellers. The theory was then inducted by Lord Rayleigh, who formulated a mathematical model delineating cavitation in an incompressible fluid. The giant advantages of sonochemistry were first revealed by Wood and Loomis in 1927. By using ultrasound, they dealt with two types of reactions successfully, such as the redox of sulfite in an aqueous solvent and accelerating some conventional reactions, for example, the hydrolysis of dimethyl sulfate, demonstrating that ultrasound is a promising tool in chemical synthesis even without its mechanism [35]. In the following decades, a surge of research gradually detected the rationale of sonochemistry mentioned before. The sonolysis of an organic liquid was achieved by Schultz and Henglein in 1953 [36]. In 1967, Lierke and his colleague inspected that metal powders could be formed when they were using ultrasound to atomize molten metals [37]. During this period, ultrasound technology was improved significantly by the update of homogenizers, causing it to be more accessible to generate high-frequency ultrasound. This led to the evolution of new applications in sonochemistry, including synthesis, polymerization, and environmental remediation.

As far as the 1980s with the progress of ultrasonic generators, researchers surprisingly found that ultrasound indicated extraordinary applications in chemistry. The term “sonochemistry” was introduced by Neppiras when he traced back acoustic cavitation.

After the 21st century, just like a gusher, a huge number of ultrasound-assisted reactions were reported, particularly, and ultrasound has been playing an increasingly significant role, especially in the synthetic chemistry process.

In 2003, Phillippe inspected vesicle deformation with a powerful ultrasonic tool [38]. Three years later, Cintas and Pedro summarized existing applications that have an eco-friendly process [39]. In 2021, Machado and his colleagues presented the enhancement of the synthesis of N- and O-heterocyclic compounds, which play an eminent role in pharmaceutical chemistry by introducing sonochemistry and recyclable heterogeneous catalysts [40].

**Figure 1:**
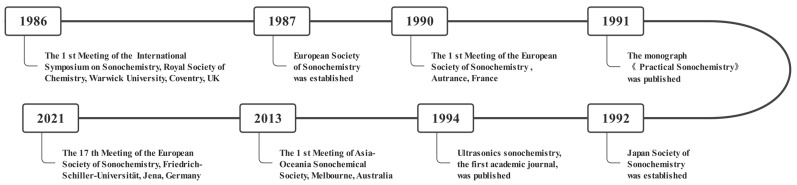
A brief history of sonochemistry conferences, associations, journals, and works.

Following the development of sonochemistry, some famous conferences, associations, journals, and works on the topic of sonochemistry have been established or published (Figure 1) [41,42].

On the basis of our preceding study, this work, which covers the research over the last two decades, will highlight recent developments in the ultrasound-assisted synthesis of imidazole-based compounds as well as the advantages they possess. Meanwhile, it is anticipated that this review will provide new opportunities for the investigation of a practical design for imidazole-containing compounds.

## 2. Development of Ultrasound-Assisted Imidazole-Based Compounds Synthesis

As the significance of green chemistry and environmentally friendly principles has gradually grown, scientists have been searching for alternative trials to enhance the traditional but cumbersome reactions. With the superior features of sonochemistry, in other words, some extraordinary advances have been acquired by applying the ultrasound-assisted technique in conventional reactions in imidazole derivatives’ synthesis, bringing surprising merits to the following reactions, including the Debus–Radziszewski reaction, the Phillip–Ladenburg reaction, the Ullmann reaction, as well as some other reactions.

### 2.1. Debus–Radziszewski Imidazole Synthesis

**Scheme 2:**
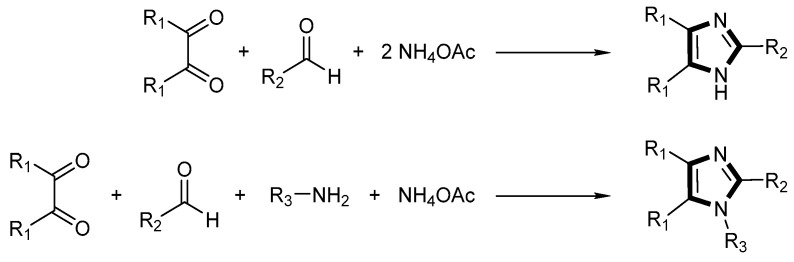
The Debus–Radziszewski imidazole synthesis.

The illustrious Debus–Radziszewski reaction, originally claimed by Debus in 1858 and enhanced by Radziszewski in 1882, has been widely used in synthesizing a series of imidazole derivatives (Scheme 2) [17,18]. Typically, the D–R reaction proceeds via the dehydration condensation of a diketone, an aldehyde, and two equivalents of ammonia. This reaction can afford 1,2,4,5-tetrasubstituted imidazoles when one equivalent of ammonia could be an alternative to the primary amine.

However, this classic method has many drawbacks, for instance, harsh reaction conditions, hazardous chemicals, expensive acid catalysts, complicated working and purification procedures, and a long reaction period, with side reactions leading to mediocre yields.

With the ultrasonic irradiation utilized in the D–R reaction, a more compact way to prepare imidazole-based compounds was discovered. The desirable advantages of those ultrasound-based conditions mentioned, such as, briefly speaking, in comparison to conventional ways that synthesize promising imidazole and its derivatives, demonstrate that a higher yield, milder conditions, and shorter reaction time are achieved while pollution and expenses are diminished.

**Scheme 3:**
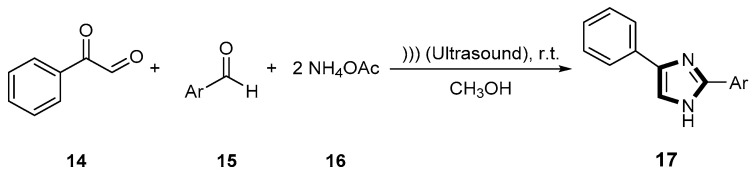
Synthesis of **17**.

Bandyopadhyay and his co-workers reported a method with the D–R process to synthesize 2-aryl-4-phenyl-1*H*-imidazoles **17** under ultrasound irradiation by the condensation of phenylglyoxal monohydrate **14**, aldehyde **15**, and ammonium acetate **16** without using any catalyst/solid support (Scheme 3) [43]. This sonicated approach proved to be milder, more rapid, and more eco-friendly compared to the traditional D–R reactions. However, the results of these reactions were not satisfactory in the yields of 57~73% at room temperature after 25~60 min, which may be attributed to the lack of an appropriate supplementary catalyst.

To improve the effectiveness of ultrasound-assisted D–R reactions, researchers have investigated modifying the conditions of D–R reactions, including using nano-catalysts, metal complexes catalysts, ionic liquids catalysts, organic catalysts, inorganic catalysts, and oxidants.

#### 2.1.1. Nano-Catalysts

**Scheme 4:**
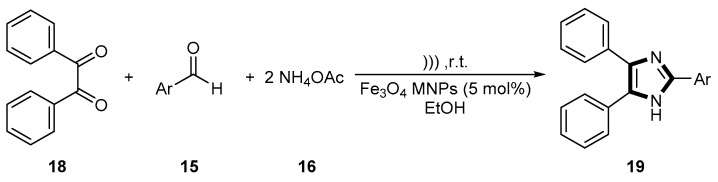
Synthesis of **19** catalyzed by Fe_3_O_4_ MNPs.

Magnetic nano-particles (MNPs) were applied for the one-pot three-component sonochemical method to synthesize 2,4,5-trisubstituted imidazoles **19** by Safari and his group in 2012 (Scheme 4) [44]. This ultrasound-assisted protocol afforded the corresponding imidazoles in high yields of up to 97% under the catalysis of Fe_3_O_4_ MNPs which was previously synthesized through the coprecipitation method. The effect of ultrasound was validated by observing significant reductions in reaction time with the assistance of ultrasound, compared to cases under the conventional condition in the absence of sonication (25~45 min for the ultrasonic condition and 120~180 min for the reflux condition). In addition, this type of reaction, catalyzed by Fe_3_O_4_ MNPs, demonstrated wide applicability to substrates.

**Scheme 5:**
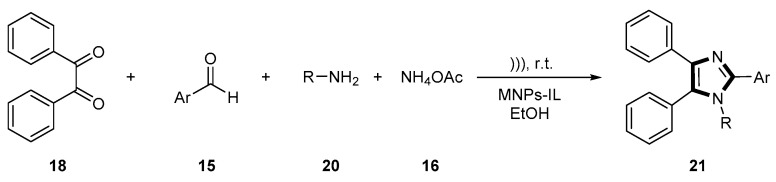
Synthesis of **21**.

One year later, they reported the methods that applied ionic liquid to support the Fe_3_O_4_ MNPs catalyst in the synthesis of multi-substituted imidazoles **21** (Scheme 5) [45]. The modified heterogeneous catalyst showed comparably great activity, as the yields in this approach could reach 95%. In addition, the MNPs-IL catalyst can be easily recycled and reused with only a 7% loss in activity after five cycles. Avoiding the use of harmful catalysts, an optimal reaction temperature, a high yield, and a simple method makes it a more effective alternative than the conditions from conventional methods.

**Scheme 6:**
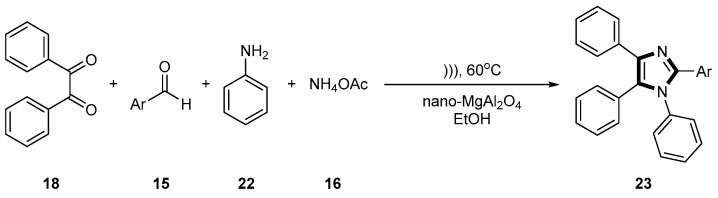
Synthesis of **23** catalyzed by nano-MgAl_2_O_4_.

In 2013, they continued to report a synthesis of 1,2,4,5-tetrasubstituted imidazoles **21** by a one-step condensation of four components of benzil **18**, aryl aldehyde **15**, ammonium acetate **16**, and aniline **22** (Scheme 6) [46]. These reactions were promoted by the function of ultrasonic irradiation and nano-magnesium aluminate spinel MgAl_2_O_4_ as a Lewis acid catalyst, affording **23** products with great effectiveness. After the assays for the optimization of the temperature and frequency of ultrasound, 60 °C and 50 kHz were determined to be the most satisfied conditions with a yield of up to 98%.

**Scheme 7:**
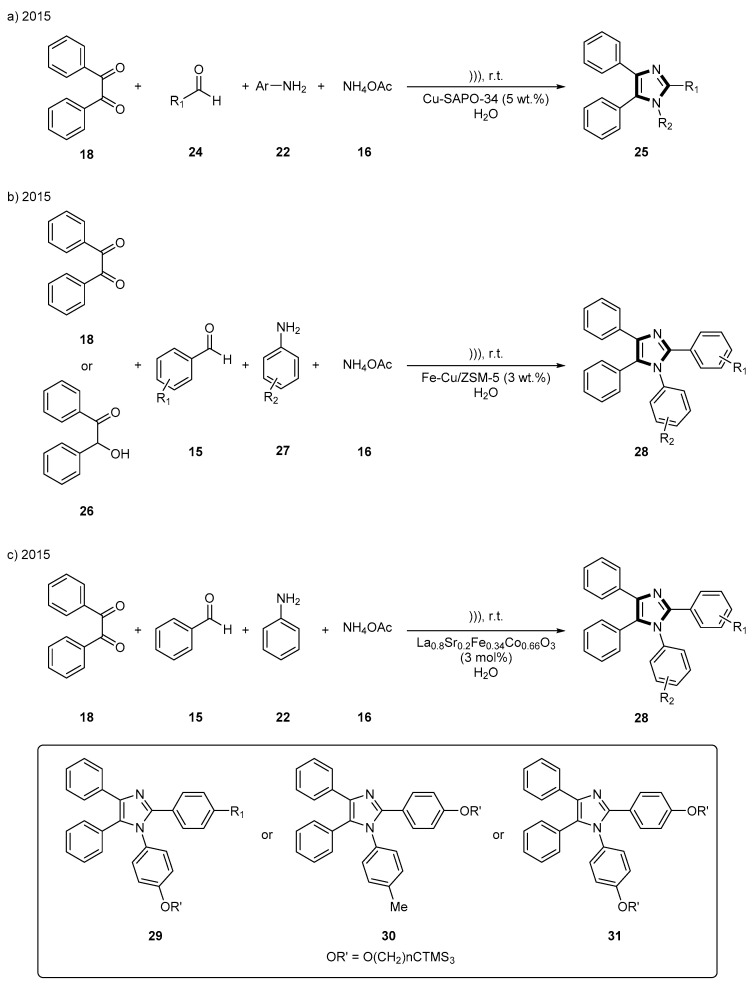
Three methods for the synthesis of **25** or **28** by the Safa group.

Safa and his co-workers published three new methods for synthesizing multi-substituted imidazoles **25** and **28** in 2015. As shown in Scheme 7, in method (a), they utilized a string of M-SAPO-34 as acidic zeolite nano-catalysts of the one-pot condensation [47]. In the evaluation of the catalytic efficiency of various kinds of M-SAPO-34 (M = Cu, Fe, Co, Mn), the best outcome was observed under the catalysis of Cu-SAPO-34, with a yield of 95% within 5 min. In method (b), the catalyst was replaced by Fe-Cu/ZSM-5, another metal-based zeolite catalyst, showing the greatest catalytic efficiency among a string of monometallic and bimetallic catalysts on the support of ZSM-5 zeolite [48]. Benzoin **26** can serve as the substrate of this process as an alternative to benzil **18**, with comparable reaction times (8 min for **26** and 2~3 min for **18**) and yields (88~93% for **26** and 97~99% for **18**). In addition, the Fe-Cu/ZSM-5 catalyst can be easily recycled without an obvious loss in activity. In further research, this approach has yielded products that are utilized to synthesize organosilicon-containing imidazole substrates **29**–**31**, offering a wide range of chemical variety and biological functions that may be valuable for novel drug development. In method (c), these researchers applied a series of catalysts (La_x_Sr_1−x_Fe_y_Co_1−y_O_3_ nano-perovskites) in the synthesis of **28** [49]. La_0.8_Sr_0.2_Fe_0.34_Co_0.66_O_3_ was reported as the most efficient one among these multi-component oxides that were prepared by the sol-gel auto-combustion. With the function of La_0.8_Sr_0.2_Fe_0.34_Co_0.66_O_3_ and ultrasound, **28** were synthesized in great yields above 92%.

**Scheme 8:**
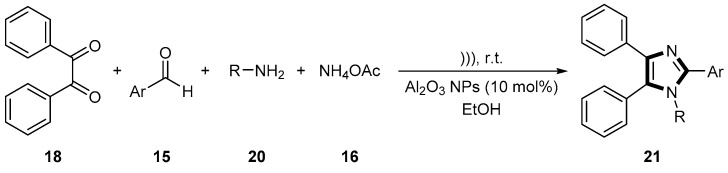
Synthesis of **21**.

In 2015, the γ-Al_2_O_3_ NPs-catalyzed method for the synthesis of highly substituted imidazoles was introduced by Reddy and colleagues (Scheme 8) [50]. They allocated γ-Al_2_O_3_ NPs to catalyze the multi-component reaction of benzil **18**, arylaldehyde **15**, amines **20**, and NH_4_OAc **16**, which afforded 1,2,4,5-tetrasubstituted imidazoles **21** with great yields of up to 95%. With the application of ultrasonic irradiation, the reactions showed higher yields and shorter times compared to the cases under traditional conditions. Moreover, Al_2_O_3_ NPs catalysts exhibited great reusability, which was validated to have comparable activities for four cycles after separation.

**Scheme 9:**
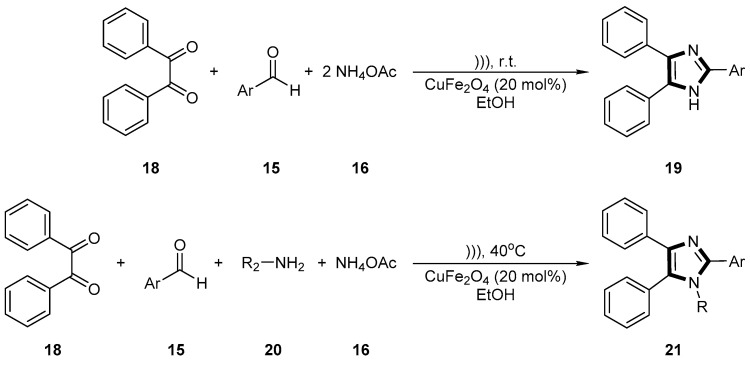
Synthesis of **19** and **21**.

In 2016, Sanasi and his co-workers introduced the application of nano-copper ferrite (CuFe_2_O_4_) with a spinel structure in the one-pot, three- and four-component synthesis of substituted imidazoles **19** and **21** (Scheme 9) [51]. This type of novel catalyst showed high efficiency, bringing great yields of about 90%. The reusability accomplished by the simple operation of the catalyst is one of the major features of this reaction.

**Scheme 10:**
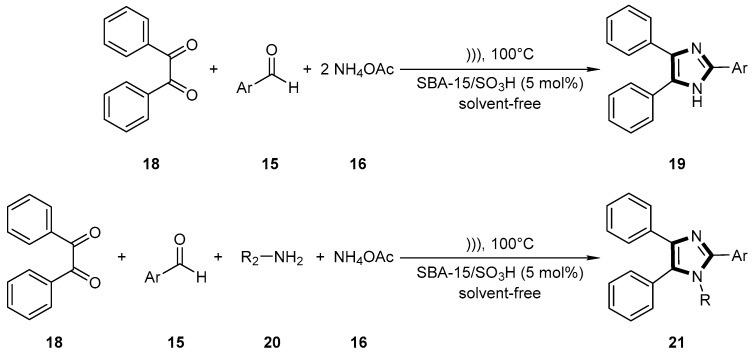
Synthesis of **19** and **21**.

Meanwhile, Doustkhah and his co-workers found a great system combining mesoporous nano-reactor SBA-SO_3_H and ultrasonic radiation, which is used to synthesize heterocycles with biologically active ones (Scheme 10) [52]. In the system, highly substituted imidazole **19** and **21** are synthesized through the multi-component coupling method. SBA-SO_3_H induced by ultrasound can accelerate the mass transfer of mesoporous remarkably, leading to great yields and short reaction periods. When Ar was phenyl, the ultrasound-assisted synthesis of **21** obtained a yield of 94% in 8 min, while the same case under the condition of high-speed stirring as the alternative to ultrasound only achieved a yield of 80%, costing 4 h. Other imidazole derivatives, **19** and **21**, were afforded in the yields of 80~92%. Notably, splendid selectivity and tolerance, and access to various functional groups are realized in this refined trial.

**Scheme 11:**
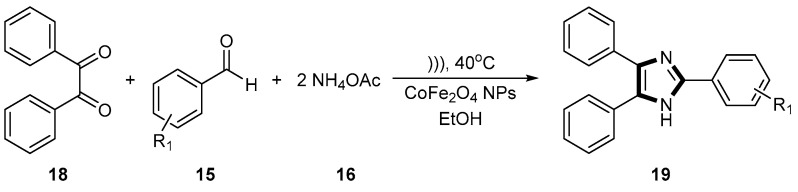
Synthesis of **17**.

Eidi and co-workers published the synthesis of 2,4,5-trisubstituted imidazoles **19** with the catalysis of CoFe_2_O_4_ NPs (Scheme 11) [53]. The spinel CoFe_2_O_4_ NPs were synthesized by the coprecipitation of Co^2+^ and Fe^3+^ in ammonia under the N_2_ atmosphere. They applied the CoFe_2_O_4_ nano-catalyst in one-step condensations of diketone **18**, aldehyde **15**, and ammonium acetate **16**, with sonication. These reactions obtained high yields of up to 95% within 20 min under the function of ultrasonic irradiation and paramagnetic nano-CoFe_2_O_4_. In addition, the catalyst exhibited good reusability in the protocol mentioned, with few losses in catalytic activity after four cycles.

**Scheme 12:**
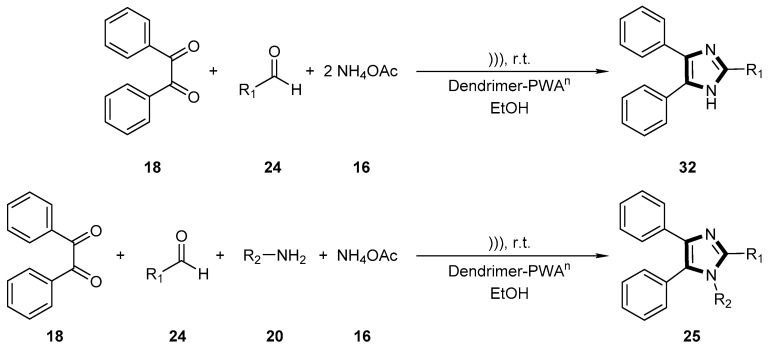
Synthesis of **25** and **32**.

In 2017, Esmaeilpour developed a green one-pot method to synthesize a string of 2,4,5-trisubstituted **32** and 1,2,4,5-tetrasubstituted imidazole **25** (Scheme 12) [54]. They prepared nano-silica dendritic polymer-supported H_3_PW_12_O_40_ NPs (Dendrimer-PWA^n^) as the reusable catalyst of the ultrasound-assisted reaction. Dendritic-PWA^n^ played the role of the potent acid catalyst that highly promoted dehydration condensation with the synergetic effect of ultrasound, leading to excellent yields of up to 95%. This approach demonstrated wide applicability to a variety of substrates as well.

**Scheme 13:**
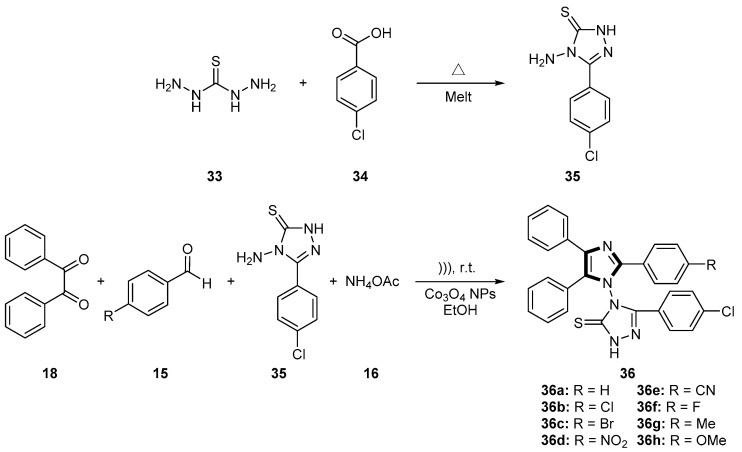
Synthesis of **36a**–**36h**.

In 2018, the Ghasemzadeh group reported a concise and efficient method to prepare Co_3_O_4_ NPs via a one-pot reaction (Scheme 13) [55]. At that point, they investigated the catalytic effects of the nano-catalyst on the one-pot reaction of synthesizing a series of 1,2,4,5-tetrasubstituted imidazole **36** using ultrasonic irradiation. Simple work-up, neutral conditions, short reaction times (12~28 min), and excellent yields (91~97%) make it a meaningful alternative to traditional procedures that synthesize biologically active imidazole.

The antimicrobial activity of imidazole compounds against some common pathogenic bacteria was studied by a paper diffusion method in vitro, such as, *Escherichia coli*, *Bacillus subtillis*, *Staphylococcus aureus*, *Salmonella Typhi*, and *Shigella dysentrae* species. The results showed that compounds **36c**, **36f**, and **36h** had the highest contents against all bacteria, compound **36a** had the highest activity against *Bacillus subtilis*, and compound **36g** possessed the highest antioxidant activity in *Salmonella dysentery*.

**Scheme 14:**
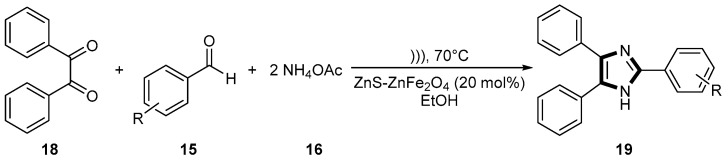
Synthesis of **17**.

Varzi and his colleagues introduced a new approach to producing mixed nano-catalyst ZnS-ZnFe_2_O_4_ in 2019 (Scheme 14) [56]. The nano-ZnS-ZnFe_2_O_4_ was synthesized through the chemical coprecipitation method and then using the Lewis acidic catalyst to promote the sonicated synthesis of **19**. The yields in this approach reached 95%, with a reaction time of 15 min. This hybrid nano-catalyst exhibited great effectiveness and recyclability and could be reused for six cycles with subtle activity loss. The reaction even obtained an 86% yield in the sixth cycle of catalyst utilization.

**Scheme 15:**
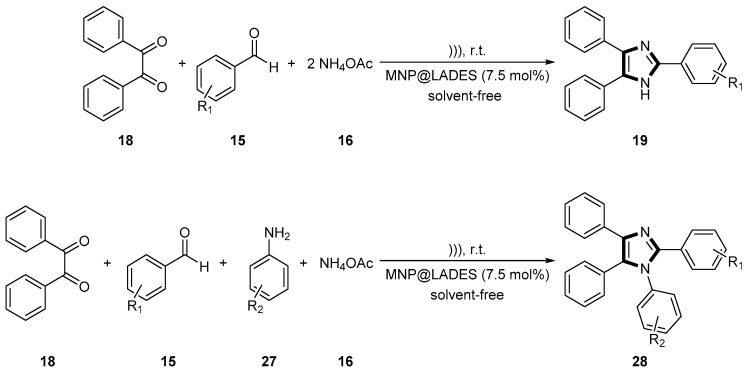
Synthesis of **19** and **28**.

At the same time, Nguyen and colleagues reported an efficient multi-component synthesis of 2,4,5-trisubstituted **19** and 1,2,4,5-tetrasubstituted imidazoles **28**, catalyzed via a magnetic nano-particle in a Lewis acidic deep eutectic solvent (MNP@LADES) (Scheme 15) [57]. For the synthesis of the catalyst, the Fe_3_O_4_ MNPs were first prepared and then coated with tetraethylorthosilicate. MNP@LADES was obtained after the intermediate product underwent the process of functionalization and a reaction with [Urea]_4_[ZnCl_2_]. Despite the laborious preparation of the catalyst, this ultrasound-assisted approach using MNP@LADES afforded substituted imidazoles in great yields of up to 94% without byproducts being observed. In addition, the catalyst can be easily recovered by magnetic separation and reused for five cycles without attrition of catalytic activity.

**Scheme 16:**
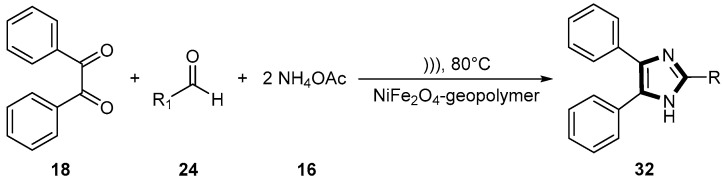
Synthesis of **32**.

In 2020, Hajizadeh and her co-workers developed a one-pot three-component reaction by using a novel and green NiFe_2_O_4_-geopolymer nano-catalyst to prepare imidazole derivatives **32**, which was accelerated by ultrasonic irradiations (Scheme 16) [58]. The nano-NiFe_2_O_4_ supported on geopolymer exhibited great catalytic effects, validated by comparison to other catalysts, including, bentonite, geopolymer, and NiFe_2_O_4_ NPs, and stable recyclability.

**Scheme 17:**
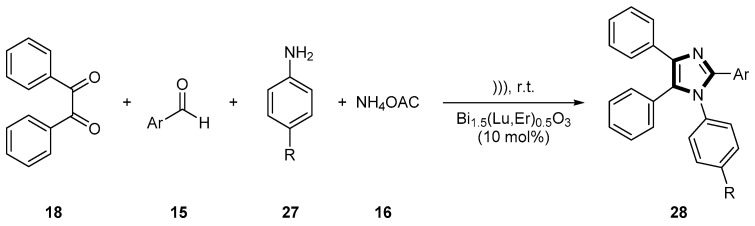
Synthesis of **28**.

One year later, Kohan et al., published the synthesis of 1,2,4,5-tetrasubstituted imidazole derivatives **28** catalyzed by a Bi_1.5_(Lu,Er)_0.5_O_3_ nano-catalyst via ultrasonic assistance (Scheme 17) [59]. This Lewis acidic heterogeneous catalyst possessed good catalytic activity and simple reusability. With the presence of nano-Bi_1.5_(Lu,Er)_0.5_O_3_ and the application of ultrasound, this method afforded imidazole-based compounds in great yields of roughly 90% within 5 min at room temperature.

**Scheme 18:**
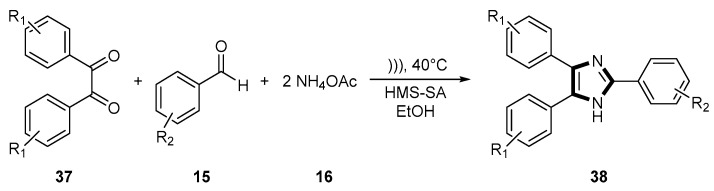
Synthesis of **38**.

Arora and his co-workers reported the one-pot synthesis of substituted imidazoles **38** with the catalysis of modified hollow magnetite spheres (HMS) catalysts under ultrasonic irradiation in 2021 (Scheme 18) [60]. They began with the synthesis of HMS, followed by the functionalization of HMS with sulfamic acid groups, forming HMS-SA. The fabricated materials, as catalysts, were introduced in the one-pot three-component synthesis of trisubstituted imidazoles **38** and could be recovered and reused with almost negligible losses in efficacy. These ultrasonic-assisted reactions afforded **38** in great yields (92~99%).

**Scheme 19:**
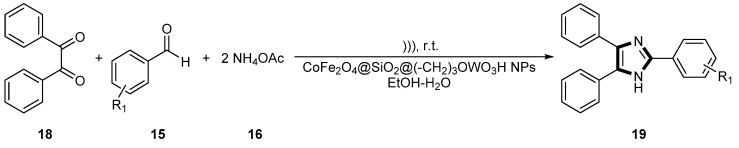
Synthesis of **19**.

In early 2023, Kermanizadeh and Naeimi reported the design and preparation of modified silica-coated cobalt ferrite nano-particles (CoFe_2_O_4_@SiO_2_@(-CH_2_)_3_OWO_3_H NPs) for the synthesis of trisubstituted imidazoles **19** (Scheme 19) [61]. Prepared via a four-step process, this novel catalyst exhibited solid stability and potent catalytic efficacy. The 2,4,5-aryl imidazoles **19** were afforded through this method in yields of up to 95% within 8 min. After reactions, the solid acid catalyst could be easily recycled and reused for five cycles with consistent activity.

#### 2.1.2. Metal Complex Catalysts

**Scheme 20:**
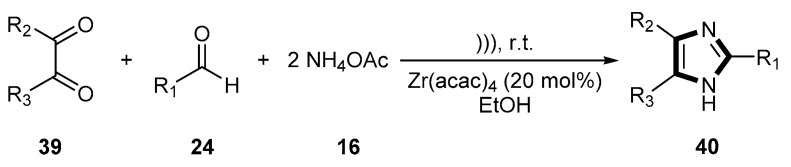
Synthesis of **40**.

In 2008, Khosropour et al., introduced a simple and green ultrasonic-assisted synthesis of 2,4,5-trisubstituted imidazole **40**, with zirconium (IV) acetylacetonate (Zr(acac)_4_) as the catalyst and with aldehydes **24**, benzils **39**, and ammonium acetate **16** as the starting materials (Scheme 20) [62]. In this approach, the best yield reached 97%. Compared to the cases under the reflux condition that took around 3 h with a maximum yield of 84%, these ultrasound-assisted reactions typically finished in 20~50 min.

**Scheme 21:**
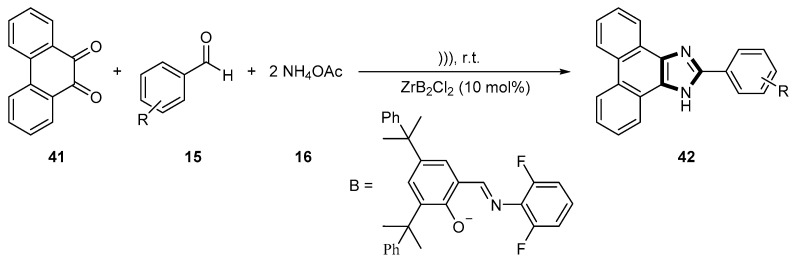
Synthesis of **42**.

In 2011, the Damavandi group reported that bis [*N*-(3,5-dicumylsalicylidene)-2,6-fluoroanilinato] zirconium(IV) dichloride was a highly interesting catalyst, using ultrasonic irradiation for the synthesis of 2-aryl-1*H*-phenanthro[9,10-d]imidazole derivatives **42** (Scheme 21) [63]. A novel one-pot method was brought out in the efficient synthesis of **42** under ultrasound with yields of up to 93%, bringing compact reaction procedures, lenient conditions, and affordable materials.

**Scheme 22:**
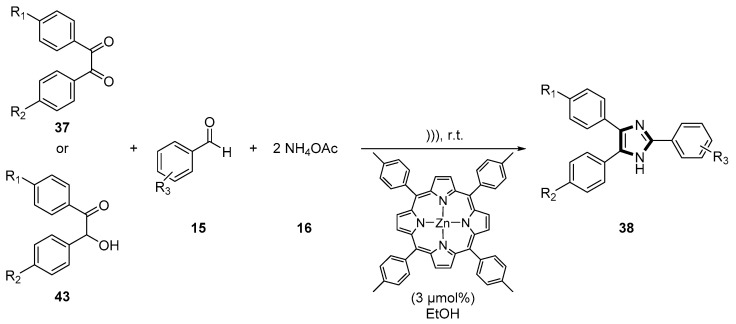
Synthesis of **38**.

In 2011, a productive one-pot procedure of the synthesis of the 2,4,5-trisubstituted imidazoles **38** was published by the Safari group (Scheme 22) [64]. The condensation reaction was catalyzed by Zinc (II) [tetra-(4-methylphenyl)] porphyrin, which is a repeatable and new product, using 1,2-diketones **37** or α-hydroxyketones **43** with aromatic aldehydes **15** and ammonium acetate **16** as the starting source. Excellent yields of 87~97% were obtained in this method.

#### 2.1.3. Ionic Liquids Catalysts

**Scheme 23:**
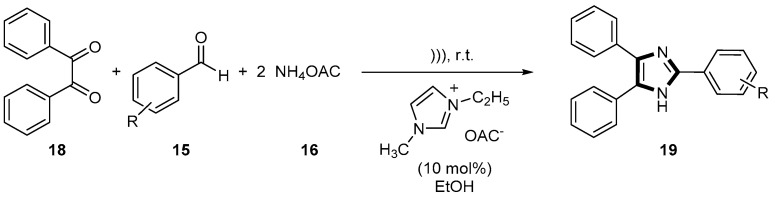
Synthesis of **19**.

In 2010, the Zang group found that the ionic liquid 1-ethyl-3methylimidazole acetate ([EMIM]OAc) is a functional catalyst for the one-step synthesis of 2-aryl-4,5-diphenyl imidazole **19** (Scheme 23) [65]. With the utilization of ionic liquid [EMIM][Oac] and ultrasound, the yields significantly increased to a maximum of 96% compared with a method involving the absence of the catalyst (15%) or ultrasonication (18%). This procedure possesses obvious advantages, such as avoiding the use of harmful catalysts or reagents, reactions at room temperature, and simple steps for the separation process.

**Scheme 24:**
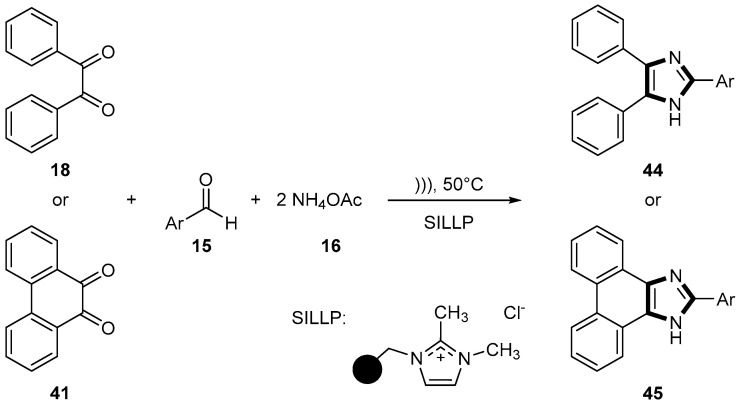

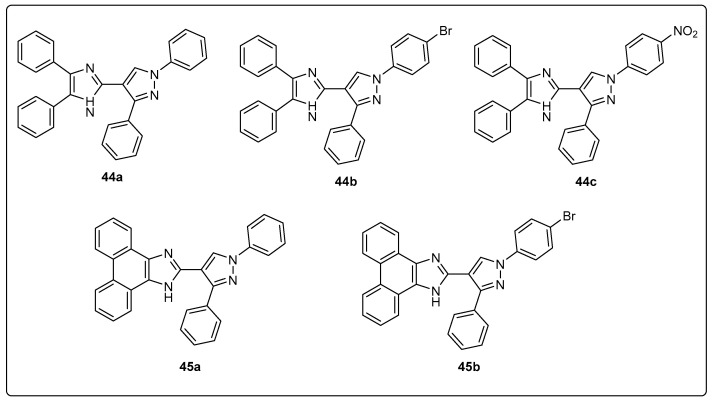
Synthesis of **44**(**a**–**c**) and **45**(**a**–**b**).

In 2013, the Saffari Jourshari group reported an interesting method for imidazole synthesis by the condensation of benzil **18** or 9,10-phenanthrenequinone **41** with aldehydes **15** and ammonium acetate **16**, catalyzed via ultrasound in an ionic liquid-like phase (SILLP) (Scheme 24) [66]. The products **44** and **45** were afforded in great yields of around 90% with a short reaction time of 3~6 min. Additionally, many of the products they synthesized exhibited potent antimicrobial activity, such as **44a**, **44b**, **44c**, and **44a**, **44b**. This perhaps needs further and future in-depth research and excavation to find novel drugs for clinical applications of great value.

**Scheme 25:**
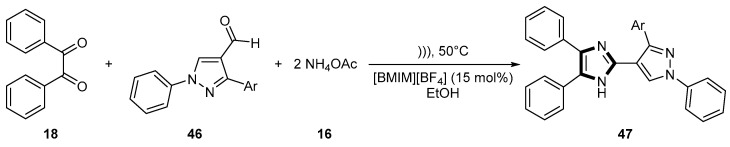
Synthesis of **47**.

After four years, 1-butyl-3-methylimidazolium tetrafluoroborate ([BMIM][BF4]), an efficient ionic liquid catalyst, was applied to synthesize imidazole compounds via ultrasonic irradiation by the Shirole group (Scheme 25) [67]. Compared to the cases of the conventional reflux condition, the yields in ultrasound-assisted reactions increased by around 10%, and the reaction time decreased by two thirds, validating the efficacy of ultrasound.

**Scheme 26:**
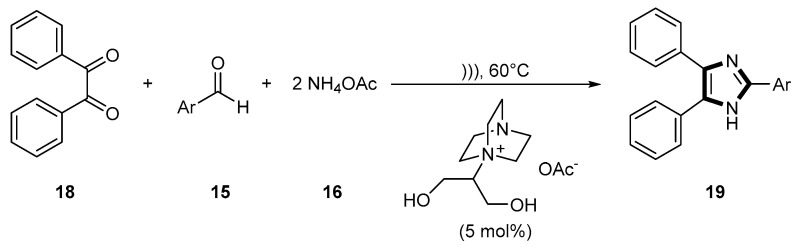
Synthesis of **19**.

In 2018, Arafa and colleagues introduced the [DABCO-DOL][OAc], a DABCO-based ionic liquid catalyst, which was a powerful and eco-friendly catalyst for this one-pot multi-component imidazole-based compounds’ synthesis (Scheme 26) [68]. Through the substitution reaction of DABCO and 2-chloro-1,3-propandiol, followed by the anion exchange, this ionic liquid catalyst was quickly prepared. Compared with traditional methods, the proposed method afforded **19** more effectively, with a simple operation and high yields of up to 99%. In addition, the ionic liquid catalyst can be reused with comparable efficacy after seven cycles.

**Scheme 27:**
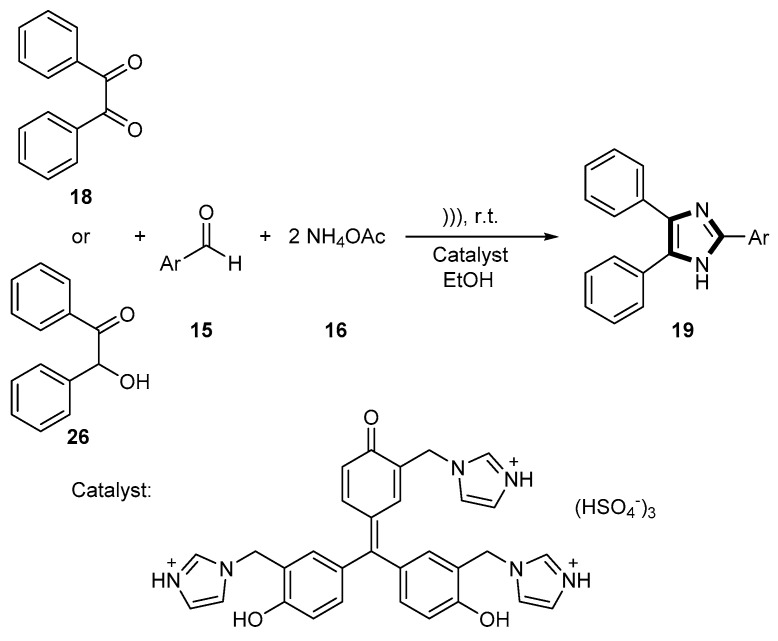
Synthesis of **19**.

In 2020, Hilal and his colleagues reported a novel acidic ionic liquid, [2-(imm)-4-{b(immh)m}c][HSO_4_]_3_, which was applied as the catalyst in the synthesis of 2,4,5-trisubstituted imidazole derivatives **19** (Scheme 27) [69]. With auxiliary ultrasonication, this ionic liquid catalyst was prepared via a five-step procedure and significantly promoted the condensation of aldehydes **15**, ammonium acetate **16**, and benzil **18**/benzoin **26**. These ultrasound-assisted reactions obtained yields of 73~98% with a reaction time of 35~60 min, while the methods under the conventional reflux condition obtained lower yields (38~86%) and a longer reaction time (120~190 min). In addition, the ionic liquid catalyst could be easily recycled for three cycles.

**Scheme 28:**
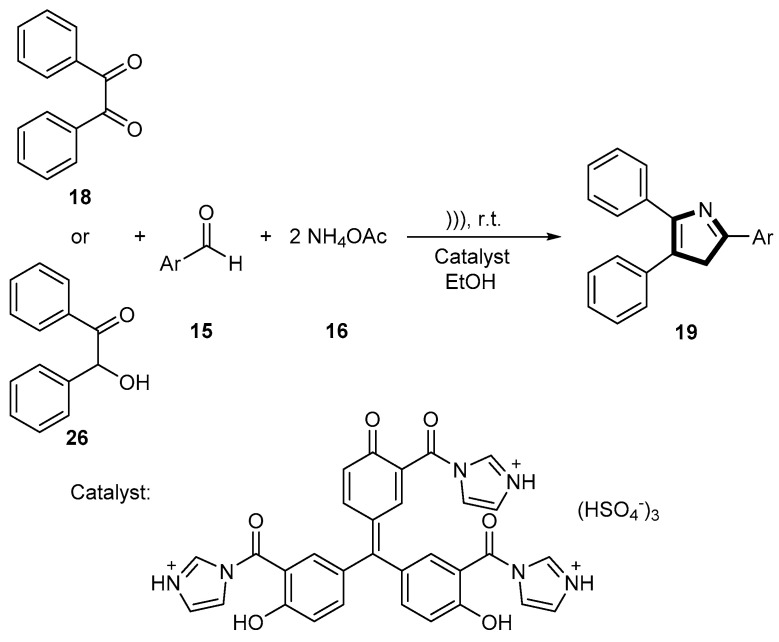
Synthesis of **19**.

In 2021, another similar ionic liquid catalyst [{(IMC)-4-OMBH}BIM][HSO_4_]_3_ was applied by Ahmed and Hanoon in the ultrasound-assisted synthesis of **19** (Scheme 28) [70]. The updated acidic ionic liquid was developed based on the structure of [2-(imm)-4-{b(immh)m}c][HSO_4_]_3_ and demonstrated comparable catalytic activity and was easier to obtain due to one less step in the preparation procedures. The [{(IMC)-4-OMBH}BIM][HSO_4_]_3_ is regarded as an eco-compatible and highly efficient catalyst under ultrasound irradiation, bringing a convenient isolation process for the products. The catalyst could be reused for five cycles with limited loss in catalytic activity.

#### 2.1.4. Organic Catalysts

**Scheme 29:**
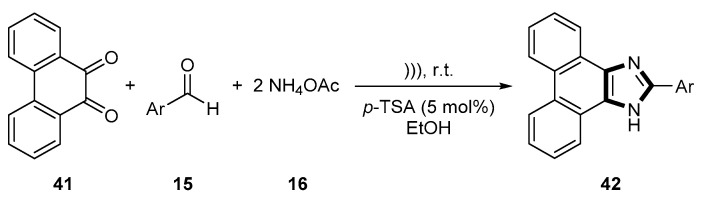
Synthesis of **42**.

In 2011, Damavandi developed a satisfactory one-step multi-component method for the synthesis of 2-aryl-1*H*-phenanthro[9,10-d] imidazoles **42** through ultrasonic irradiation (Scheme 29) [71]. To investigate the most suitable catalyst for the condensation of aldehydes **15**, 9,10-phenanthrenequinone **41,** and ammonium acetate **16**, researchers examined several organic acids and their salts. The highest yield (94%) was obtained under ultrasonic irradiation and the catalysis of *p*-toluenesulfonic acid (*p*-TSA), using EtOH as the solvent. This *p*-TSA-catalyzed approach offered a simple and efficient way to synthesize **42** with the facile operation to purify.

**Scheme 30:**
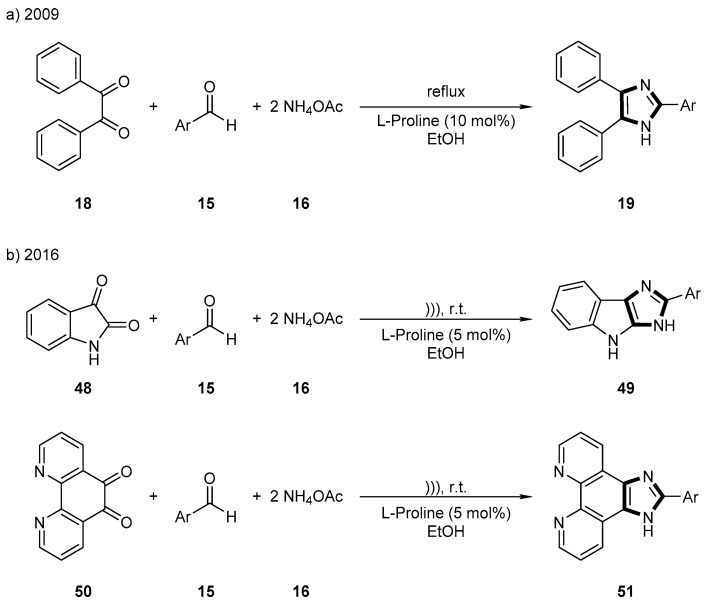
Synthesis of **49** and **51**.

Five years later, this group continued to use L-proline as the catalyst, which is a convenient and user-friendly reagent with great catalytic activity. Previous research published by Shitole et al., in 2009 reported the application of L-proline in the standard D–R reaction, in which L-proline served as a bifunctional catalyst containing both a basic secondary amine group and an acid carboxylic group (Scheme 30a) [72]. These reactions obtained great yields (75~94%) but cost several hours (120~300 min) to undergo complete progression. Furthermore, in 2016, Damavandi and his co-worker developed the L-proline-catalyzed one-pot synthesis of 3,4-dihydro-2-arylimidazo[4,5-b]indole **49** and 2-aryl-1*H*-imidazo[4,5-f][1,10]phenanthroline **51** under ultrasonic irradiation, starting by aromatic aldehydes **15**, indoline-2,3-dione **48** or 1,10-phenanthroline-5,6-dione **50**, and ammonium acetate **16** (Scheme 30b) [73]. It is worth noting that the reaction time significantly decreased to around 15 min due to the utilization of ultrasound, with comparable yields of 74~96%.

**Scheme 31:**
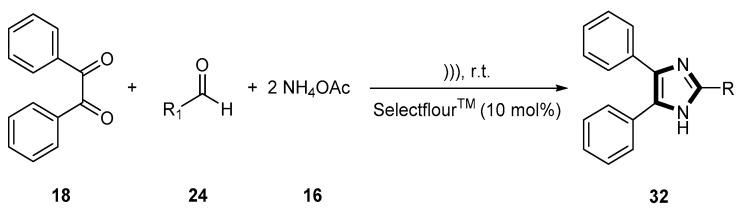
Synthesis of 32.

In 2014, the Heravi group developed an interesting one-pot three-component routine to synthesize 2,4,5-trisubstituted imidazoles **32**, using ultrasound irradiation without a solvent in the presence of Selectfluor™ (Scheme 31) [74]. The Selectfluor^TM^ served as the Lewis acidic catalyst in the reaction that activated the carbonyl group, effectively promoting the three-component condensation under sonication. These ultrasonic-assisted reactions offered **32** in great yields of 82~99% within 3~15 min.

**Scheme 32:**
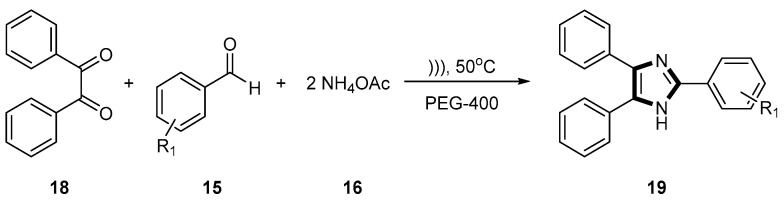
Synthesis of **19**.

Devkate and his co-workers claimed an enthralling one-pot three-component reaction that synthesizes 2,4,5-triaryl-1*H*-imidazole **19** via a novel approach, in 2017 (Scheme 32) [75]. They utilized polyethylene glycol PEG-400 as an effective and recoverable catalyst to promote the condensation of the benzil **18**, aromatic aldehydes **15**, and ammonium acetate **16**. Compared to the traditional reflux condition, every reaction using auxiliary ultrasound afforded product **19** higher yields and a shorter reaction time. The yields have risen significantly for most reactions (87~95% for the ultrasonic method and 67~75% for the conventional method) and remarkably reduced the reaction time (8~15 min for the ultrasonic method and 53~80 min for the conventional method).

**Scheme 33:**
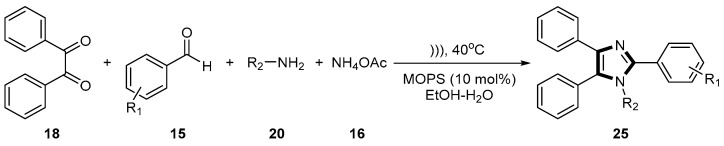
Synthesis of **25**.

In 2020, Khandebharad and his co-workers reported an eco-compatible procedure to synthesize tetrasubstituted imidazole-based compounds **25** in which 3-(*N*-morpholino)propane sulfonic acid (MOPS) was utilized as a green and efficient acidic catalyst (Scheme 33) [76]. With the effect of ultrasonic irradiation and MOPS, the one-pot four-component condensation of benzil **18**, aldehydes **15**, primary amine **20,** and ammonium acetate **16** afforded product **25** in great yields within a short reaction time. Notably, the MOPS catalyst could be recycled and reused for three cycles. According to their statistics, the majority of the yields were promoted from approximately 80% to 90% and the reaction time was roughly halved from 20~60 min to 10~30 min with the help of ultrasound.

**Scheme 34:**
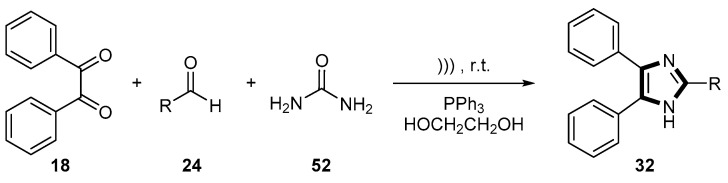
Synthesis of **32**.

In the same year, the Behrouz group reported the triphenylphosphine(PPh_3_)-catalyzed ultrasound-assisted reaction to synthesize 2,4,5-trisubstituted imidazole **32** efficiently (Scheme 34) [77]. They found that PPh_3_ exhibited great catalysis activity in the D–R reaction at room temperature, which provided an effective and eco-friendly method with a cheap and harmless catalyst for the preparation of **32**. Disparate from other typical D–R reactions, these researchers employed urea **52** as the nitrogen source, resulting in higher yields (up to 95%) compared to the assays of NH_4_OAc (up to 87%). In this approach, imidazole derivatives **32** were obtained in excellent yields of 80~95%.

#### 2.1.5. Inorganic Catalysts

**Scheme 35:**
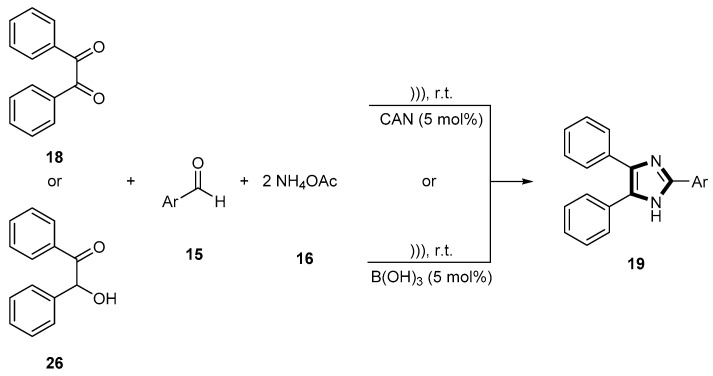
Synthesis of **19**.

In 2009, Shelke and his co-workers synthesized the structurally diverse 2,4,5-trisubstituted imidazole **19** via the one-pot three-component method, catalyzed by non-toxic ceric (IV) ammonium nitrate (CAN), assisted by ultrasound, benzil **18**/benzoin **26**, aldehydes **15,** and ammonium acetate **16** as the starting material (Scheme 35) [78]. CAN exerted its catalysis activity as a Lewis acid that activated the carbonyl. In that year, they continued to utilize boric acid as the acidic catalyst in the synthesis of 2,4,5-triaryl imidazole derivatives **19** under sonication [79]. Boric acid with the supplementary ultrasound irradiation ameliorated the three-component condensation with great effectiveness, offering an efficient and convenient protocol for the preparation of imidazole-based compounds with yields above 85%.

**Scheme 36:**
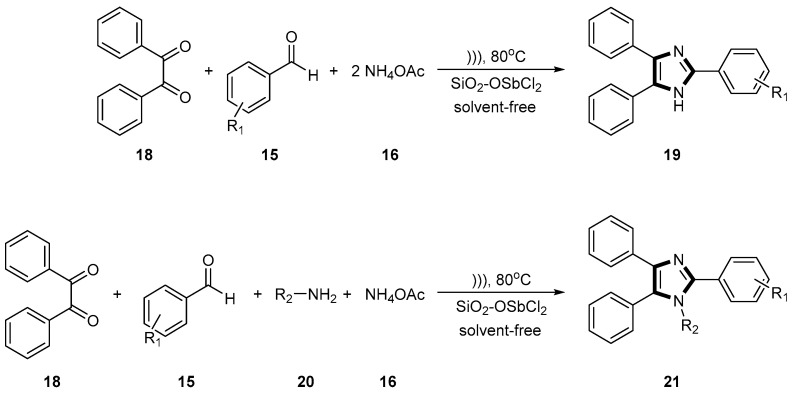
Synthesis of **19** and **21**.

In 2014, Safari and his co-workers formulated a one-pot synthesis of substituted imidazoles based on the ultrasound method and the SiO_2_-OSbCl_2_ catalyst (Scheme 36) [80]. The synthesis of 2,4,5-trisubstituted imidazoles **19** and 1,2,4,5-tetrasubstituted **21** via a multi-compound condensation was accelerated by using assisted ultrasonication and antimony(III) chloride with the support of silica gel (SiO_2_-OSbCl_2_) as a Lewis acid catalyst, under a solvent-free condition. This protocol demonstrated great applicability to various substrates with comparable effectiveness. Most reactions obtained great yields of above 80% with a short reaction time (10~33 min).

**Scheme 37:**
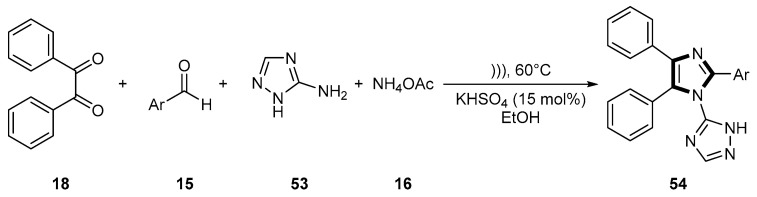
Synthesis of **54**.

In 2020, Dastmard and her co-workers reported a one-pot, four-component method using acidic KHSO_4_ as an effective catalyst under ultrasonic irradiations to synthesize 4,5-diphenyl-1*H*-imidazol-1-yl-1*H*-1,2,4-triazole derivatives 54 (Scheme 37) [81]. The yields were obtained in 70~96%, and the reaction time ranged from 10 min to 25 min. Based on the results from trials in Scheme 37, they continued to sift several bioactivity compounds after they obtained the results. The antimicrobial activities of compounds that they synthesized against Gram-negative bacteria (*Escherichia coli.*, *Pseudomonas aeruginosa.*) and Gram-positive bacteria (*Bacillus subtilis.*, *Micrococcus luteinis.*) were screened below. They evaluated the compounds’ antioxidant activities and then found that many of the products have promising potential regarding antibacterial activity and high antioxidant activity.

#### 2.1.6. Oxidant

**Scheme 38:**
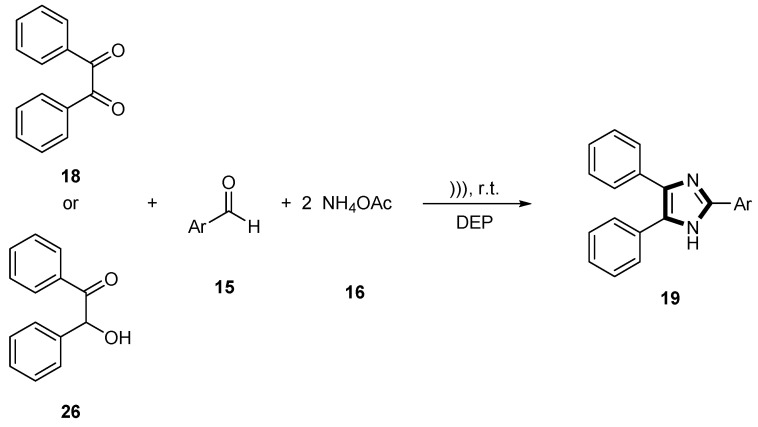
Synthesis of **19**.

In 2012, the Nagargoje group used diethyl bromophosphate (DEP) as the oxidant for a one-pot three-component condensation to obtain 2,4,5-triaryl-imidazole compounds **19** (Scheme 38) [82]. The oxidant agent DEP enabled benzoin to serve as a feasible alternative to benzil as the substrate of the D–R reaction, and the cases of both benzoin and benzil afforded the product **19** in great yields (91~97%) under an ultrasound-assisted condition.

### 2.2. Phillips–Ladenburg Imidazole Synthesis

**Scheme 39:**
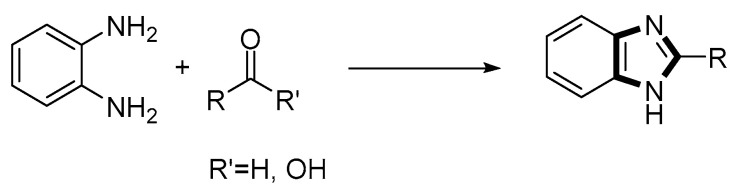
The Phillips–Ladenburg imidazole synthesis.

In 1875, Ladenburg reported the synthesis of benzimidazole via the condensation of 1,2-diaminobenzens (1,2-DAB) and aldehydes [19,20]. Around 1928, Phillips enhanced the Ladenburg method by using carboxylic acids as substitutes for aldehydes [21,22,23]. Scheme 39 shows the typical type of the Phillips–Ladenburg reaction that affords benzimidazoles from 1,2-DAB. The conventional Phillips reaction usually requires harsh reaction conditions, for example, a combination of high temperature (170 °C) and microwave irradiation, which, definitely suffers high-energy consumption, an expensive apparatus, and verbose reaction times.

Therefore, ultrasound-assisted methods are introduced by researchers, bringing atom economic and eco-compatible effects to those conventional ones. Ultrasonic irradiation illustrates the P–L reaction, fueling higher yields, operating simplicity, and production efficiency.

**Scheme 40:**
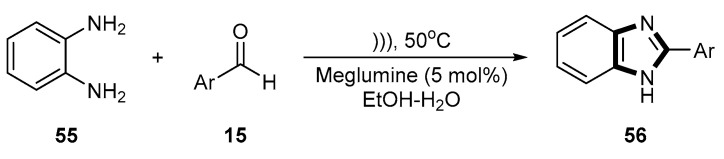
Synthesis of **56**.

In 2019, Nongrum and her co-workers, with ultrasonic assistance, brought about a green approach toward the fabrication of benzimidazole scaffolds **56** (Scheme 40) [83]. These researchers used meglumine as the green and harmless catalyst for the Phillips–Ladenburg reaction. The effect of ultrasound was evaluated by comparing it with the cases under the condition of reflux which resulted in a longer reaction time (5 h for reflux stirring and 25~30 min for ultrasound) and lower yields (50~68% for reflux stirring and 80~90% for ultrasound).

**Scheme 41:**
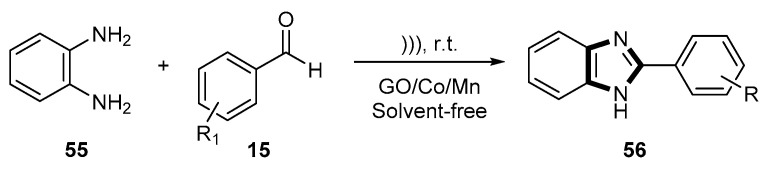
Synthesis of **54**.

In 2020, Karami and his co-workers reported a novel nano-catalyst, Co/Mn supported by GO (Graphene oxide) which was prepared by using metal oxide as a carrier under ultrasound irradiation (Scheme 41) [84]. This reusable nano-catalyst has been used to synthesize some benzimidazole from corresponding aldehydes and 1,2-phenylene-diamine **56**. Compared with applying a thermal condition at 80 °C, the ultrasonic way only required room temperature to undergo a reaction with comparable yields.

**Scheme 42:**
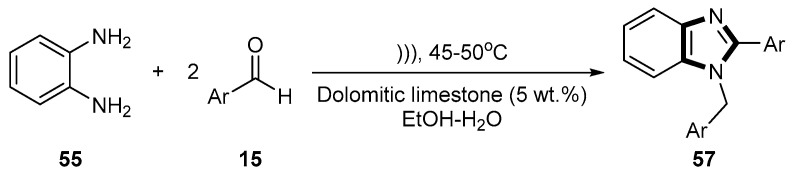
Synthesis of **57**.

In the same year, Godugu and his colleagues claimed an environmentally benign protocol to synthesize **57**, with the ancillary ultrasound and natural dolomitic limestone catalyst, which was utilized as a heterogenous for the Philips reaction (Scheme 42) [85]. They surprisingly found that by employing ultrasound as well as the catalyst, standout refinements were acquired, such as non-toxic catalysts, a short reaction time (10~15 min), excellent yields (94~98%), and an uncomplicated isolation of the products.

**Scheme 43:**
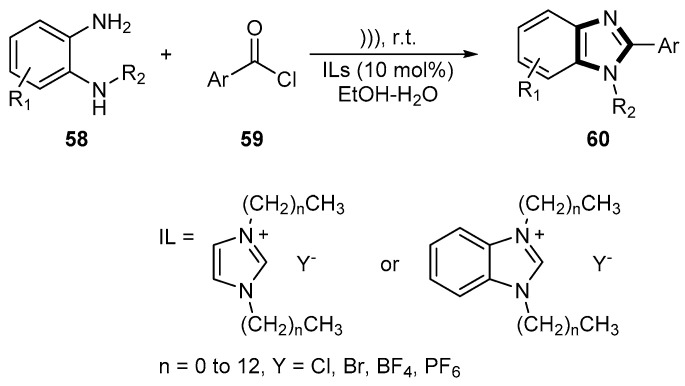
Synthesis of **60**.

In 2022, Meeniga et al., emaciated an environmentally benign ionic liquid for the precursors of the synthesis of 2-aryl benzimidazoles **60** under ultrasonication (Scheme 43) [86]. The application of imidazole- and benzimidazole-based ionic liquids as the catalyst of the Phillips–Ladenburg reaction resulted in a brief reaction time (2~10 min), good yields (67~99%), and a great tolerance for various substrates. Compared to the conventional reaction, they sought a method that corresponds with green chemistry principles.

### 2.3. Ullmann-Type Reaction

**Scheme 44:**
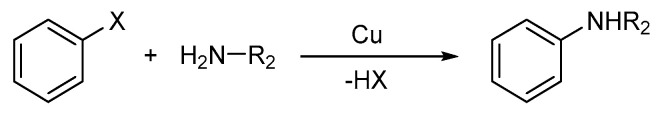
The Ullmann-type reaction.

The Ullmann reaction is a broadly used method for carbon-nitrogen bond-forming claimed by Ullmann in 1904 (Scheme 44) [87]. Though limited by large-timespan and high-energy-cost traditional reaction conditions (requiring copper for the catalyst and a high temperature of more than 180 °C), it is widely used for the synthesis of some compounds such as imidazole. Though it was widely applied in labs, the classic way shows annoying downsides, such as complex procedures, high pollution, and expensive materials.

**Scheme 45:**
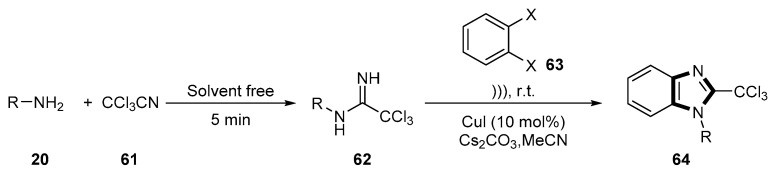
Synthesis of **64**.

In 2019, Nematpour and her co-workers developed an alternative reaction routine for the novel synthesis of 2-(trichloromethyl)-benzimidazole **64** under ultrasound irradiation, with the aminetrichloroacetonitrile **62** adduct and 1,2-dihalo benzene **63** as the starting materials (Scheme 45) [88]. This improved Ullmann-type reaction only has one-pot, copper-catalyzed, and three-component conditions, offering a series of merits including more affordable raw materials, a short reaction time (30~35 min), and high yields (72~94%).

### 2.4. Other Imidazole Synthesis

**Scheme 46:**
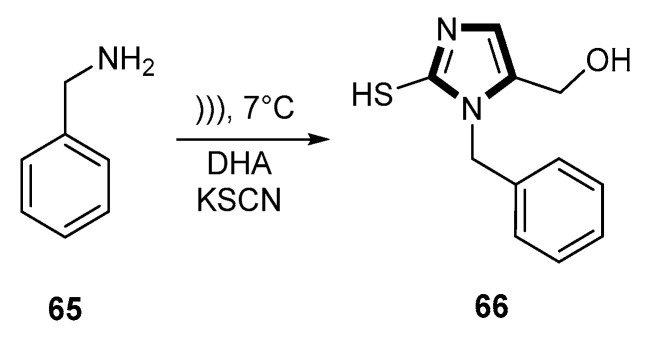
Synthesis of **66**.

In 2008, Entezari and his co-workers delved into the synthesis of 5-hydroxymethyl-2-mercapto-1-benzylimidazole **66** with an ultrasound-assisted trial (Scheme 46) [89]. They manipulated the conditions such as temperature and vapor pressure of the solvent in order to optimize the yields of the reactions, reaching a yield of 90% after half an hour at 7 °C, while the yields of the traditional trials reached only 70% after 72 h. Apparently, applying the ultrasound-assisted procedure properly could achieve a high yield of the product.

**Scheme 47:**
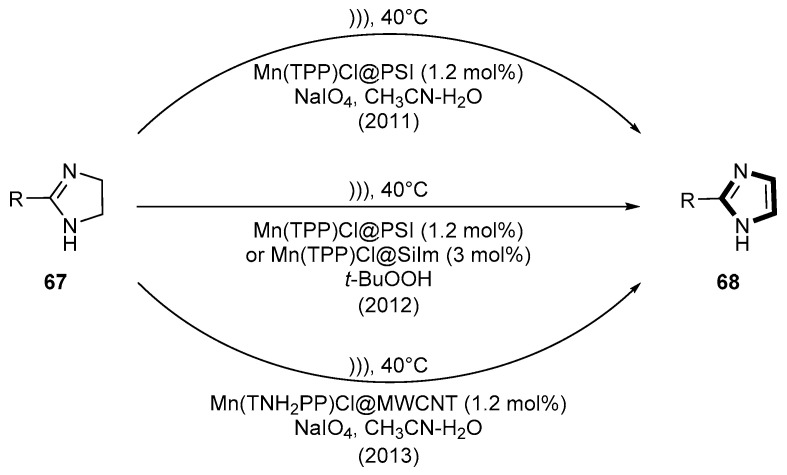
Synthesis of **68** via ultrasound-assisted dehydrogenation.

In 2011, the Kargar group reported the synthesis of 2-substituted imidazole **68** via the dehydrogenation of imidazolines [90]. Under the catalysis of [Mn(TPP)Cl@PSI], the oxidizing agent NaIO_4_ effectively promoted the dehydrogenation of 2-substituted imidazolines **67** to form the corresponding product **68**. This ultrasonic method afforded **68** in high yields of 74~94% after 1 h, while the same cases under magnetic stirring obtained comparable yields but cost 10 h. In 2012, they continued to develop tetraphenylporphyrinatomanganese(III) chloride, [Mn(TPP)Cl], as the catalyst of the oxidation process. In those methods, **67** was oxidated by *t*-BuOOH with great effectiveness via ultrasonication in the presence of Mn(TPP)Cl supported on PSI or SiIm [91]. With a reaction period of 1 h, the yields of the Mn(TPP)@PSI- and Mn(TPP)Cl@SiIm-catalyzed reactions were 68~90% and 75~95%, respectively. In the next year, researchers applied [Mn(TNH_2_PP)Cl@MWCNT] as the modified catalyst and NaIO_4_ as the new oxidant in the dehydrogenation of 2-substituted imidazolines **67** [92]. The yields for this approach ranged from 71% to 93%. In these three catalytic systems, a variety of 2-imidazoline compounds were effectively converted to the corresponding imidazoles, and all these catalysts can be recycled five times without an undesirable loss in activity. Along with making use of ultrasonic irradiation, complex procedures were simplified, and pollutants were diminished as well as energy was conserved, while yields were increased, and the reaction time was reduced under sonication (Scheme 47).

**Scheme 48:**
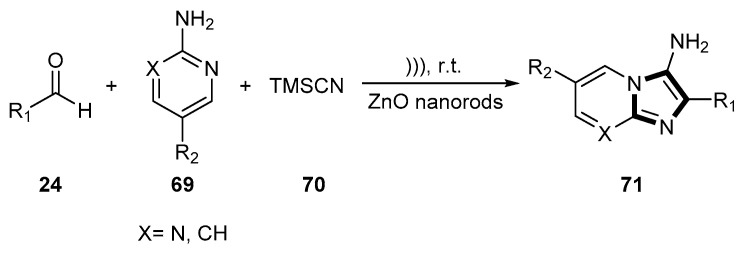
Synthesis of **71**.

In 2013, Sadjadi and Eskandari published a novel approach to synthesize imidazo[1,2-a]azine compounds **71** (Scheme 48) [93], taking the aldehydes **20**, trimethyl-silylcyanide (TMSCN) **70**, 2-aminopyrimidine or 2-aminopyridine **69** as the starting material. The imidazole scaffold was constructed with significant efficiency via a three-component condensation, facilitated by ultrasonic irradiation and the catalysis of ZnO nano-rods that had been previously prepared from the decomposition of Zn(OAc)_2_·2H_2_O. Compared to the cases under conventional conditions, the method applying ultrasound irradiation received higher yields (83~90% for ultrasound, 65~76% for reflux, and 70~80% for stirring) and a shorter reaction time (7~12 min for ultrasound, 20~35 min for reflux, and 20~40 min for stirring). The ZnO nano-rod catalyst was able to be reused for three cycles. The reactions using the recycled catalyst obtained great yields of 88%, in addition to the typical features of ultrasound-assisted reactions, including a fast reaction time, a simple operation, and eco-compatibility.

**Scheme 49:**
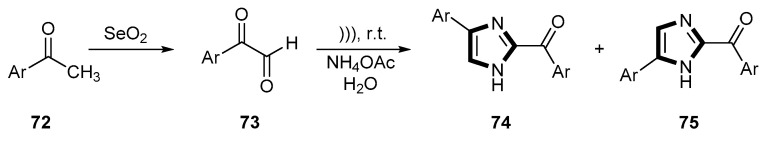
Synthesis of **74** and **75**.

In 2014, Khalili and Rimaz offered ultrasound promotion to the synthesis of (4 or 5)-aryl-2-aryloyl-1*H*-imidazoles **74** and **75**, which were formed by the self-condensation reaction of arylglyoxal hydrates **73** in the presence of ammonium acetate, using water as solvent under irradiation by ultrasound (Scheme 49) [94]. The precursor could be derived via the oxidation of acetophenones utilizing SeO_2_. The application of sonication led to higher yields and shorter reaction periods compared to the conventional method. The ultrasound-assisted reactions obtained yields of 72~95% in 4 min, while the cases without ultrasound obtained yields of 55~86% after 45 min.

**Scheme 50:**
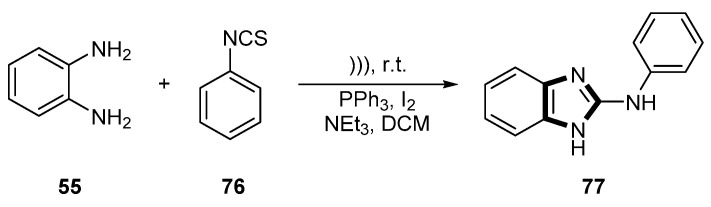
Synthesis of **77**.

In 2016, Phakhodee and his co-workers claimed ultrasound could be applied in the synthesis method of substituted 2-aminobenzimidazoles **77** (Scheme 50) [95]. Benzene-1,2-diamine **55** and phenyl isothiocyanates **76** were coupled to create the intermediate mono-thiourea, which was similar to the Phillips–Ladenburg reaction. The intermediate then underwent cyclo-desulfurization via the function of PPh_3_-I_2_ system and was converted into the product *N*-aryl-2-aminobenzimidazoles **77**. This process was accelerated by ultrasonic irradiation, leading to higher efficiency in both time (10~25 min) and yields (76~94%). In addition, benzene-1,2-diamine can be replaced by other similar compounds such as 2-aminophenol in this reaction, which provides a novel method of the construction of 2-amino benzoxazoles and other relative frameworks.

**Scheme 51:**
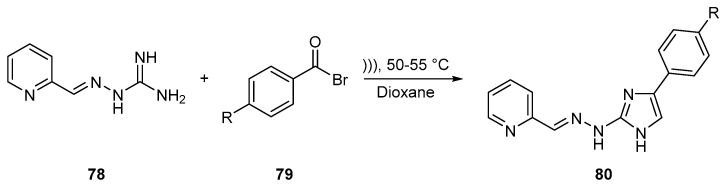
Synthesis of **80**.

Three years later, Sreenivasulu et.al., published the preparation of pyridine-linked hydrazinylimidazoles **80** (Scheme 51) [96]. This part of the guanidyl group in the substrate can react with 4-substituted benzoyl bromine **79**, forming the imidazole heterocycles under the irradiation of ultrasound. In comparison to the classic thermal method, the utilization of sonication resulted in a dramatically more rapid reaction (36~52 min for ultrasound and 300~540 min for reflux) and higher productivity (80~92% for ultrasound and 63~71% for reflux). Moreover, the majority of the products exhibited antimicrobial efficacy in the activity testing, indicating that these compounds could serve as an inspiration for the development of novel antibacterial or antifungal drugs.

## 3. Conclusions

As mentioned, imidazole derivatives play a pivotal role in pharmaceutical, organic, and material chemistry, commensurately boosting a thriving desire for both laboratories and industrial companies. However, the conventional methods suffer significantly since they demonstrate a relatively low yield and are time-costly for most reactions, contrasted with the ultrasound-assisted protocols. The ultrasound-assisted synthesis, which meets the requirement of green chemistry and mitigates the above problems, has attracted more and more researchers’ attention.

Sonochemistry, as a nascent technique, demonstrates surprising advantages in the synthetic process, serving as a dramatical solution to those drawbacks mentioned in traditional reactions. Over the past two decades, plenty of new trials applying ultrasound to imidazole synthesis have been published, of which the majority were modified using ultrasonic irradiation on the basis of the classic conventional named reactions. These ultrasound-assisted modified syntheses exhibit excellent promise for the application of synthesis of imidazole compounds, with milder conditions, greater yields, and more significantly, higher atom economy and better eco-compatibility that conform to the principles of green chemistry.

In this review, we comprehensively traced back the enhancement of imidazole synthesis with the ancillary function of ultrasound. In the future, however, the enhancement of some ultrasonic reactions is not remarkable. Their reaction conditions should be further optimized, and more suitable reaction conditions under ultrasound-assisted synthesis, such as temperature, catalyst, oxidant, etc., should be explored or searched for. In addition, other typical imidazole syntheses based on ultrasound-assisted methods have not been reported yet. The optimization for the catalyst with a simpler structure and wider substrate tolerance needs more focus in future directions.

In summary, the ultrasound-assisted technique is able to enhance efficacy and selectivity and reduce cost and pollution. Ultrasound-assisted imidazole synthesis has shown its potential to innovate the field of synthetic chemistry by providing more efficient, eco-friendly, and sustainable approaches to heterocyclic compound synthesis.

## Figures and Tables

**Table 1:** Biological or pharmaceutical activities of several imidazole-based compounds.

Function	Structure	References
Anticancer	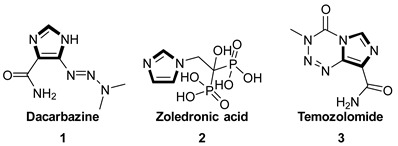	[7,8,9]
Antifungal	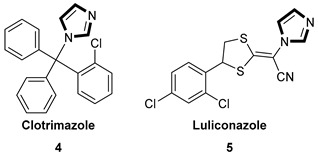	[10,11]
Antibacterial	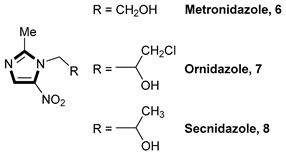	[12]
Antiparasitic	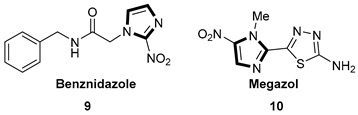	[13,14]
Analgesic	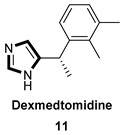	[15]
Fungicide	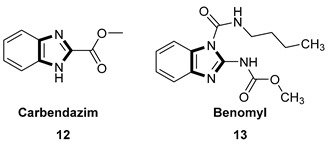	[16]

## Data Availability

Not applicable.

## Sample Availability

Not available.
